# 老年晚期非小细胞肺癌姑息治疗获益研究进展

**DOI:** 10.3779/j.issn.1009-3419.2015.07.11

**Published:** 2015-07-20

**Authors:** 姗彤 蒋, 萍萍 李

**Affiliations:** 100142 北京，北京大学肿瘤医院暨北京市肿瘤防治研究所中西医结合暨老年肿瘤科 Key Laboratory of Carcinogenesis and Translational Research (Ministry of Education), Department of Integration Medicine and Geriatric Oncology, Peking University Cancer Hospital and Institute, Beijing 100142, China

**Keywords:** 肺肿瘤, 老年肿瘤, 姑息治疗, Lung neoplasms, Elderly cancer, Palliative care

## Abstract

在中国，肺癌的发病率和病死率均居恶性肿瘤的第一位。有近一半的肺癌发生在年龄大于70岁的老年患者。肺癌中约85%是非小细胞肺癌(non-small cell lung cancer, NSCLC)，且大多数肺癌患者发现时已属中晚期。老年晚期NSCLC患者具有伴随疾病多、器官功能衰退等特点。制定恰当的治疗策略是我们面临的挑战。姑息治疗作为特殊的医疗关怀，是老年晚期NSCLC的重要治疗方式之一。低剂量局部姑息放疗可以有效改善老年NSCLC患者的呼吸道症状，且副作用可以耐受；对于表皮生长因子受体(epidermal growth factor receptor, *EGFR*)突变的老年晚期NSCLC，吉非替尼在疾病控制率、症状缓解等方面均可使老年患者从治疗中获益。同时，老年晚期NSCLC患者对厄洛替尼也显示出良好的耐受性；氩氦刀在治疗老年NSCLC患者的应用有增加趋势且患者对氩氦刀技术的耐受性和反应性都较好。中医药在改善临床症状、减少放化疗的毒副作用和提高生活质量方面有较好的疗效；社会心理支持疗法在一定程度上可缓解NSCLC患者的困扰，但其系统性有待完善。姑息治疗的评估和介入时机，是患者能否从中获益的重要因素。文章介绍了老年NSCLC姑息治疗获益研究的进展，为老年NSCLC的姑息治疗提供依据。

## Abstract (en)

Lung cancer is the leading cause of death among all cancers in China. It also has the highest incidence when compared to other cancers. Almost half of all lung cancers occur over 70-year-old. Approximately 85% of all lung cancers are non-small cell lung cancer (NSCLC). The majority of patients are advanced lung cancer. Due to the unique alterations in physiology, elderly patients are at a greater risk of toxicity from chemotherapy. Palliative care as a special medical care is an important treatment for elderly patients with advanced NSCLC. Low-dose palliative radiotherapy can improve respiratory symptoms in elderly patients with NSCLC, with the tolerated side effects. Elderly patients with epidermal growth factor receptor (*EGFR*) mutation can benefit from gefitinib and have a good tolerate of erlotiib. Cryocare Surgical System has an increasing trend of application in the treatment of elderly patients with NSCLC. Chinese medicine has effects in improving clinical symptoms and reducing side effects of chemotherapy, it can also improve the quality of life in these patients. Psychosocial support therapy can alleviate the burden of patients with NSCLC to some extent, but needs to improve its systematicness. Assessment and the time of palliative care are two important factors which determine the outcome of patients. We introduce the progress in palliative care benefit of elderly NSCLC, in order to provide the basis for palliative care of elderly NSCLC.

## 1 老年肺癌发病概况和治疗特点

世界卫生组织国际癌症研究署(International Agency for Research on Cancer, IARC)2014年发布的GLOBOCAN2012癌症报告显示：2012年全球肺癌新发病例预测约180万例，死亡约160万例，分别占恶性肿瘤新发病例及死亡病例的13%及19.4%，居恶性肿瘤第一位^[1]^。我国肺癌的发病率和病死率一直占所有肿瘤的第一位，据2014年发布的有关2010年我国肿瘤发病率和死亡率的统计数据^[2]^显示，全国恶性肿瘤发病率为235.23/10万(男性：268.65/10万；女性：200.21/10万)死亡率为148.81/10万(男性：186.37/10万；女性：109.42/10万)。其中肺癌发病率为46.08/10万，占所有恶性肿瘤的19.59%；死亡率为37/10万，占所有恶性肿瘤死亡的24.87%。肺癌的预后不佳，约85%的患者确诊时已是晚期。美国国立癌症研究所(National Cancer Institute, NCI)数据^[3]^显示，将近60%新诊断的恶性肿瘤和全癌死亡的70%都是65岁以上人群。我国老年恶性肿瘤患者中60岁-69岁占16.5%，70岁-79岁占68.9%，80岁以上占14.6%^[4]^。在老年肺癌的所有类型中，NSCLC占了75%-80%^[5]^。

随着年龄老化，老年人的器官在功能调节、结构等多方面出现不同的衰退现象，使得老年人在患病时病理、体征及治疗等方面有其特殊性。老年肺癌患者同时具有老年人和肺癌的双重特点，可体现在多个不同的方面：①合并症多，如呼吸系统疾病、心血管疾病和糖尿病等，有时可合并三种以上疾病；②身体机能：老年人全身反应性降低，难以承受常规手术及放化疗的毒副反应；③心理状态和生存目标：与年轻患者相比，老年患者对心理上的关怀需求和子女们的重视度有更多的期望。他们的生存目标，特别是年龄超过85岁的老年人，更多的是少受痛苦，改善生存质量，不为子女增添负担^[6]^。一项356例老年晚期NSCLC患者的调查研究^[7]^显示，约52%(185/356)的患者自愿选择姑息治疗，包括姑息性放疗和/或化疗。因此，尽管肺癌的发生与年龄有关，且有报道证实系统性化疗在提高老年肺癌患者短期生存率、症状控制和生活质量(quality of life, Qol)方面有一定的益处，但在老年肺癌患者的治疗决策上，系统性化疗方案在临床上选择依然较少^[6, 8, 9]^。这也进一步说明老年肺癌治疗的特殊性。所以我们首先应根据患者的功能状态、合并疾病状态及预期寿命对其进行评估，然后才能制定恰当的治疗策略，使老年肿瘤患者从中获益。

## 2 肿瘤姑息治疗的目标及临床指征

姑息治疗作为一种特殊健康关怀，关注疼痛和其他症状的有效控制，并按照患者和(或)家属的需要、价值观、信仰和文化提供社会心理与精神帮助。姑息治疗的目标是预防及减轻痛苦，提供所能达到的最佳生存质量，而不受疾病分期或其他治疗的限制。美国国家综合癌症网络(National Comprehensive Cancer Network, NCCN)肿瘤姑息治疗指南(2014版)^[10]^提出通过筛查应进行干预的临床指征：①难以控制的症状；②肿瘤诊断和/或治疗中出现的中-重度不适；③合并严重的身心疾病；④生命预期小于6个月；⑤转移性实体瘤；⑥患者/家属关心疾病病程及决策；⑦患者/家属有姑息治疗特殊要求。

姑息治疗在肿瘤诊治过程中扮演重要角色。在明确肿瘤诊断以后，即开始姑息治疗筛查评估。对手术、放疗、化疗等不良反应采取预防措施，对诊断时已中晚期无治愈可能的患者，采用姑息性手术、放疗、化疗、介入治疗、中西医结合以及心理支持等综合手段缓解肿瘤及尚未控制的症状，改善生存质量，延长无症状生存期。并在姑息治疗专业医生指导下，组织多学科团队针对患者情况制定个体化姑息治疗方案，以期最大程度地减轻患者的痛苦。

## 3 晚期非小细胞肺癌(non-small cell lung cancer, NSCLC)姑息治疗获益研究

早在1999年，美国波士顿市Dana Farber癌研究所的有关人员通过对917例Ⅲ期-Ⅳ期结肠癌或NSCLC患者的研究，发现选择攻击性治疗的患者存活的时间并不比接受姑息治疗的患者长。研究结果同时显示这些患者死亡的可能性是选择姑息治疗患者的1.6倍^[11]^。Temel等^[12]^报道了一项对晚期NSCLC患者进行早期姑息治疗干预的研究结果。研究共入组151例新诊断为转移性NSCLC的患者，所有患者被随机分到早期姑息治疗联合标准抗肿瘤治疗组和单纯标准抗肿瘤治疗组。分别用癌症治疗的肺功能评估量表[Functional Assessment of Cancer Therapy-Lung (FACT-L) Scale]和医院焦虑抑郁量表[Hospital Anxiety and Depression Scale]对干预后12周的生活质量(quality of life, QoL)和情绪进行评估。12周后，151例患者中，27例死亡，107例(86%, 107/124)完成试验。研究表明早期接受姑息治疗的NSCLC患者较单纯标准治疗组QoL有所提高、有较少的抑郁症状(16% *vs* 38%, *P*=0.01)且中位总生存期(overall survival, OS)也延长了2.7个月(11.6个月*vs* 8.9个月，*P*=0.02)。此研究表明早期姑息治疗的介入不仅可以提高进展期NSCLC患者的生存质量，同时可使患者生存获益。该项研究已产生重要影响。

## 4 老年NSCLC患者姑息治疗的干预手段

### 4.1 姑息性放疗

为了改善老年NSCLC患者的呼吸道症状，提高QoL。Langendijk等^[13]^对平均年龄65岁的65例患者进行姑息性放疗。入组的患者因有前哨淋巴结或远处转移等不能切除，且之前未进行过化疗和/或放疗治疗。以3 Gy/组(每周4次)，最高累计剂量为30 Gy对患者原发肿瘤及纵隔扩大到2 cm的区域和锁骨上淋巴结及两侧的纵隔淋巴结区域进行姑息性放疗。治疗过程中未对肺组织密度进行测量。以呼吸道症状的改善及维持或改善QoL为主要观测指标。以欧洲癌症研究和治疗组织(European Organization for Research and Treatment of Cancer, EORTC)问卷(QLQ-C30和QLQ-LC13)为依据，对患者放疗后2周、6周和3个月的QoL进行评价。结果显示：①症状的缓解率：咯血79%、手臂/肩膀痛56%、胸壁疼痛53%、咳嗽49%、呼吸困难39%、疲劳22%和食欲减退11%；②QoL评价：整体Qol评分在37%的患者中得到改善。量表中5个功能子量表的改善率分别是躯体功能35%、角色功能35%、认知功能46%、情感功能57%和社会功能42%。试验发现，对肿瘤治疗有反应(以肿瘤缩小至少50%为标准)的患者在症状缓解和Qol提高方面有着更显著的变化。以上研究说明，对晚期NSCLC患者进行姑息性放疗可在一定程度上缓解肿瘤，有效改善症状，提高QoL，患者可以从姑息性放疗中获益。Lonardi等^[14]^对48例晚期无法手术、有症状的NSCLC的患者进行放射治疗。其中43例患者以1.8 Gy/d·次-2 Gy/d·次剂量进行治疗，4例患者以2.5 Gy/次共进行12次-14次治疗。总体放射治疗的中位剂量是50 Gy，照射范围是肿瘤原发病灶和纵隔(1 cm-1.5 cm)。试验结束后共有47例患者进行评估，根据世界卫生组织标准，21/47(44%)缓解，17/47(35%)病情稳定，9/47(21%)病情进展。咳嗽、呼吸困难、疼痛和咯血症状有明显的改善。OS方面，48%为6个月，23%为12个月，10%为24个月，中位OS为5个月。该试验研究证实OS的长短与放射治疗的剂量在安全范围内呈现正相关。Cross等^[15]^报告，以8.5 Gy/次^*^2次的低剂量放射治疗症状性老年NSCLC患者，治疗后呼吸困难、胸痛、厌食/恶心、咳嗽、声嘶、咳血、吞咽困难的症状均得到不同程度的改善，未出现治疗相关的食管炎、肺炎和放射性骨髓炎等不可耐受的副作用。

### 4.2 靶向药物治疗

#### 4.2.1

表皮生长因子小分子酪氨酸激酶抑制剂(epidermal growth factor receptor -tyrosine kinase inhibitor, EGFR-TKI)

##### 4.2.1.1 吉非替尼

吉非替尼是一种EGFR-TKI，是治疗晚期*EGFR*突变的NSCLC一线药物。Takahashi等^[16]^分析了老年进展期NSCLC患者一线应用吉非替尼的获益情况。研究入组了20例存在EGFR突变的老年晚期NSCLC患者，患者中位年龄是79.5岁。250 mg/d的剂量给予吉非替尼直到疾病进展。以缓解率为主要终点，次要终点是生存率、安全性和Qol。结果发现：总有效率为70%(95%CI: 45.7%-88.1%)，疾病控制率为90%(95%CI: 68.3%-98.7%)。中位无进展生存期(progression-free survival, PFS)和OS分别为10.0个月和26.4个月。癌症治疗、肺癌量表功能评估(Functional Assessment of Cancer Therapy-Lung Cancer Symptom Scale, FACT-LCS)在吉非替尼治疗开始4周后显著改善(*P*=0.037)且在12周的评估期内保持稳定。FACT-LCS的7个项目中，咳嗽和气短在治疗4周后显著改善(分别*P*=0.046和*P*=0.008)。最常见的不良反应为皮疹和肝功能障碍。全程未发生治疗相关的死亡。同样为了评估老年晚期NSCLC患者应用吉非替尼治疗的结果以及对QoL的影响，来自日本东北部的研究小组^[17]^回顾分析了几项临床研究共71例年龄在70岁以上、体力状况评分(performance status, PS)在0分-2分应用吉非替尼治疗的晚期NSCLC患者，同时有34例使用卡铂联合紫杉醇治疗的对照组。主要观察指标是PFS、OS和应答率(response rate, RR)以及不良事件的发生率和Qol恶化的时间。结果显示：吉非替尼治疗组和标准化疗组相比，中位PFS(14.3个月*vs* 5.7个月，*P* < 0.001)和总RR(73.2 % *vs* 26.5%，*P* < 0.001)，吉非替尼组均优于标准化疗组，而中位OS没有显著的不同(30.8个月*vs* 26.4个月，*P*=0.42)。天冬氨酸转氨酶和/或丙氨酸转氨酶升高是最常见的不良事件(18.3%)，发生1例治疗相关的死亡(肺炎)。从以上两项研究可以看出，老年进展期NSCLC一线应用吉非替尼是有效、安全的。

##### 4.2.1.2 厄洛替尼

Jackman等^[18]^的研究指出：高龄(年龄≥70岁)肺癌患者对于厄洛替尼(erlotinib)具有良好的耐受性，常见的不良反应为皮疹和腹泻，肺癌的相关症状如咳嗽、疼痛呼吸困难等明显改善，中位OS可达10.9个月。针对厄洛替尼在老年晚期NSCLC患者中应用的一项研究^[19]^分别入组了9, 907例和9, 651例患者进行厄洛替尼安全性和有效性分析。安全性分析组： < 75岁，*n*=7, 848；75岁-84岁，*n*=1, 911；≥85岁，*n*=148；有效性分析组： < 75岁，*n*=7, 701；75岁-84岁，*n*=1, 815；≥85岁，*n*=135。研究结果显示： < 75岁、75岁-84岁和≥85岁三个亚组的中位PFS分别是65 d、74 d和72 d。证明了厄洛替尼在老年晚期NSCLC患者中应用的安全性和有效性不低于年轻患者，且无明显的毒副作用，患者耐受力较好。陈等^[20]^的研究观察了厄洛替尼在老年NSCLC脑转移治疗中的临床应用，研究入组中位年龄为73岁的32例患者。观察近期临床疗效和毒副作用。结果显示所有患者的临床缓解率为90.6%，临床受益率(完全缓解+部分缓解+稳定)为71.9%。毒副作用主要是皮疹、腹泻、痤疮和皮肤干燥等。并且得出*EGFR*突变、腺癌、女性、不吸烟的老年肺癌患者选用厄洛替尼临床获益较多的结论。

#### 4.2.2 抗血管生成药物

贝伐珠单抗是一种以血管内皮生长因子(vascular endothelial growth factor, VEGF)为靶点的单克隆抗体，可以与以铂类为基础的一线化疗药物联合使用。有两项研究提到贝伐珠单抗在老年肿瘤患者中的应用。其中一项入组了304例老年肿瘤患者，以顺铂和吉西他滨为基础化疗联合或不联合贝伐珠单抗，结果显示：老年肿瘤患者的总体生存率并未因联合使用贝伐珠单抗而改善^[21]^。Ramalingam等^[22]^报道卡铂和紫杉醇联合或不联合贝伐珠单抗治疗70岁以上的老年NSCLC患者也得到了相似的结果：联合使用贝伐珠单抗的患者总体生存率没有明显的差异(非联合组*vs*贝伐珠单抗联合组：11.3个月*vs* 12.1个月)。此研究还发现与年轻NSCLC患者相比，老年NSCLC患者白细胞减少、出血和蛋白尿的发生率较高。

### 4.3 氩氦刀治疗

氩氦靶向冷冻治疗(氩氦刀)是一项技术精确的治疗肿瘤方法。随着技术的成熟，氩氦刀在老年NSCLC患者治疗中的应用也逐渐增多。2007年，周等^[23]^利用计算机断层扫描(computed tomography, CT)引导氩氦刀对35例中位年龄为71岁的老年NSCLC患者进行靶向消融，术后患者肿瘤体积明显减少，患者的耐受力也较好。Hu^[24]^的研究也证实了这一点。2010年，Song等^[25]^以同样的方法对60例老年NSCLC患者进行治疗观察也得到了同样的结论，此研究中对治疗后的副作用进行了详细记载和分析，结果发现术后咳嗽和咯血的发生率较高(45%)，但都为轻度，进行对症处理后均得到改善。Fu^[26]^在应用氩氦刀治疗老年NSCLC的安全性分析及肺功能影响的观察中也证明了氩氦刀治疗效果理想且安全性较高，患者耐受性较好等优点。在联合应用方面，有研究^[27, 28]^证实，长春瑞滨；NP、GP、TP(长春瑞滨、吉西他滨、紫杉醇分别联合顺铂)化疗方案前进行氩氦刀靶向处理均可明显提高化疗药物的疗效，延长老年NSCLC患者的生存期，提高生存质量。而对于以氩氦刀治疗为主的老年NSCLC患者，手术前后应用生脉注射液、参芪扶正注射液、参附注射液、参芪扶正胶囊、健脾益肾颖粒等中成药辨证论治调整患者机能，可减少微创氩氦刀手术治疗的不良反应发生率，提高患者的生存质量。北京中医药大学的左明焕^[29]^以苓甘五味姜辛汤治疗50例中位年龄为67岁的氩氦刀冷冻术后发生咳嗽的老年NSCLC患者，评价方法：咳嗽及临床体征消失，内伤咳嗽在2周以上未发作者为临床治愈；咳嗽减轻，痰量减少为好转；症状无明显改变为未愈。结果观察到服药3 d，50例患者中治愈39例，占78%；好转9例，占18%，总有效率96%。充分说明氩氦刀和中医药联合应用对老年晚期NSCLC患者是一项有益的姑息治疗方法。

### 4.4 中医药治疗

游等^[30]^采用中药汤药加中成药静脉滴注对31例老年晚期NSCLC患者进行治疗，中药汤剂辩证用药：①阴虚内热证方用沙参麦冬汤加减；②脾虚痰湿证用六君子汤合二陈汤加减；③气阴两虚证用四君子汤合沙参麦冬汤加减；④气滞血瘀证用复元活血汤加减。老年患者多肾气亏虚，配合下述中药：肾阳虚用仙灵脾、仙茅、巴戟天等；肾阴虚用熟地、枸杞子、女贞子等；肾精不足用熟地黄、制首乌、黄精等；肾气不固以金匮肾气汤加减使用。辨病用药：常选清热解毒、化痰散结之抗癌中药，如七叶一枝花、白花蛇舌草、半枝莲、石上柏、干蟾皮、山慈菇、夏枯草等。根据患者情况随症加减。每日一剂，28 d为1个周期，观察服用2个周期后疗效。静脉滴主使用华蟾素注射液，稀释后滴注，连续应用14 d，间歇应用14 d，连续治疗两个周期观察。采用中医原发性肺癌症状分级量化表，症状积分改善率(%)=(治疗前积分-治疗后积分)/治疗前积分分]×100%评价疗效。≥70%为显效；30%-69%为有效；治疗前后症状积分无变化，甚至升高或症状积分改善率 < 30%为无效。结果显示：中医组显18例，有效9例，无效4例，总有效率87.1%。有研究^[31]^入组115例老年肺癌患者，分别以TP方案(紫杉醇+卡铂)和TP方案联合加味参芪汤对对照组(52例)和治疗组(63例)进行治疗。证明加味参芪汤在对老年肺癌患者化疗产生的并发症有明显的改善作用。为评价益肺败毒方维持治疗晚期NSCLC的临床疗效及安全性。刘等^[32]^将符合纳入标准的晚期NSCLC 60例随机分为治疗组(益肺败毒方组)和对照组(培美曲塞组)各30例。以4周为一个治疗周期，连续治疗两个周期。观察并随访记录两组病例的PFS、中医证候、QoL、CD^+4^CD^+25^调节性T细胞、成本效果比、毒副反应。结果显示：治疗后治疗组在延长PFS方面与对照组无差异；治疗组在改善中医证候(改善率：60% *vs* 23.3%)、QoL(提高率：53.3% *vs* 13.3%)、免疫功能方面均优于对照组。治疗组未出现药物不良反应。该研究提示：中医药维持治疗晚期NSCLC安全有效且价格低廉。杨等^[33]^回顾分析了121例老年NSCLC患者应用消癌平注射液治疗的效果，年龄：70岁-85岁，平均74.21岁。消癌平注射液治疗后，患者的QoL较治疗前有所提高(卡式评分：71.25±8.15 *vs* 76.30±5.17, *P* < 0.05)；中医临床症状改善情况：治疗后症状显著改善9例(占7.44%)，改善78例(占64.46%)，无改善34例(占28.10%)，症状总体改善率为71.90%(87/121)，与治疗前比较有统计学意义(*P* < 0.01)。提示消癌平注射液对老年晚期NSCLC患者的症状改善有一定疗效。

辨证论治是中医重要的核心理论，在姑息治疗症状控制中体现最为突出。也是中西医结合的切入点。随着临床研究的深入开展，将提供更多的姑息治疗证据.

### 4.5 社会心理支持干预

社会心理支持是重要的姑息治疗干预手段。小组或个体干预均可以提高患者的机体功能，减少心理的消极症状^[34]^。有研究证明社会心理支持干预可以提高晚期肿瘤患者的生存^[35-37]^，但是同时也有研究表示患者并未从此干预措施中获益^[38-40]^。Movsas等^[41]^报道NSCLC患者可以在接受社会心理支持中获益。2013年Gustafson等^[42]^在^Cancer^报道发表了一项针对NSCLC患者的网络在线系统姑息治疗的随机研究。研究中涉及到了包括网络信息获得，在线交流和相关辅导的一个全面增强健康综合支持系统(Comprehensive Health Enhancement Support System, SHESS)，这是一个旨在关注NSCLC患者信息支持互助的系统。该系统具体包括：①提供肺癌的良好组织管理、照顾护理和对家属亲人丧亲之痛梳理等的信息；②作为同行、专家、医生和用户之间沟通交流的平台；③从用户收集有效信息，依据相关决策规则提供反馈；④完善综合支持系统信息共享作用：如根据建议和相关信息整合，总结出一套程序化的方法或步骤，以方便共享应用^[43]^。试验中共有285例患者，患者被随机配对分到标准治疗组联合CHESS组和联合可选择的关于肺癌咨询的互联网(如：www.lungcanceralliancer.org, www.lungcanceronline.org等)培训组。培训可以使用或进行规范训练。观察时间25个月或死亡后13个月间对家属的随访，培训员通过互联网进行1次/2月的随访调查。以患者的症状困扰为终点指标，通过使用改良的埃德蒙顿症状评估量表进行评估。结果显示：在第4个月和第6个月时，使用CHESS组的患者症状困扰较比使用互联网训练组有显著的减轻。在第2个月和到第8个月的时候，二者无明显差异。进一步探索分析显示二者在对患者生存的影响上也无差异。此项研究说明对NSCLC患者采用系统性的社会心理支持干预，可缓解患者的症状困扰，并有一定时效性。

## 5 老年肿瘤患者姑息治疗获益的影响因素

姑息治疗已成为癌症综合治疗中不可或缺的完整部分，姑息治疗的目标是预期，预防和减少痛苦，对疼痛或其他痛苦症状进行有效控制并提供可能达到的最好的Qol。同时依据患者/家属的要求，提供社会心理和精神关怀。有研究证明，姑息治疗可以提高医疗质量，避免医疗资源的浪费^[44, 45]^。根据NCCN指南，姑息治疗应在肿瘤开始治疗前尽早介入。然而，临床往往更多的是在患者具有不可控制的症状或需要住院治疗之后才开始考虑姑息治疗^[46, 47]^。直到疾病的最后才开始姑息性治疗已被证明是不能够改变肿瘤患者的生存质量的^[48, 49]^。一项法国的多中心研究^[50]^回顾分析了姑息治疗在进展期NSCLC中的应用情况。研究中强调了一些晚期NSCLC常见症状的管理，如疼痛和营养不良等。研究分析了514例患者，结果发现，所有症状的姑息治疗干预效果均不理想，对疼痛和营养支持的干预相对较低，而比较频繁的是对社会心理的干预和临终关怀。从明确诊断到开始姑息治疗的时间较长，平均为35 d。决定早期姑息治疗的因素只有PS，往往PS好的患者开始姑息治疗较晚，只有当出现需要红细胞生成素(erythropoietin, EPO)、肠外营养指佂和/或转入临终关怀病房之后才开始。另外也有一项关于临终关怀的回顾性研究证明了这一点^[51]^。

由此可见，姑息治疗介入的时机，掌握评估方法和选择干预手段，是老年患者特别是老年晚期肿瘤患者能否从姑息治疗中获益的重要影响因素。NCCN老年肿瘤指南指出，老年肿瘤患者管理的挑战是：对寿命预期减少和耐受力减弱的人群，评估治疗的预期获益是否大于风险。无疑，评估治疗获益与风险是第一步也是关键。改善Qol和使老年患者生存获益的治疗必须建立在疾病情况，生理状态，患者意愿基础上的个体化治疗。而降低Qol和无明显生存获益的治疗是应该避免的。因此指南提出对老年肿瘤患者要进行老年综合评估，帮助医生调整治疗计划，指导干预措施使患者更好的耐受治疗。

由于老年患者较少纳入临床试验，目前仍然缺乏证据资料指导老年肿瘤患者的治疗。即使已报道的老年NSCLC患者姑息治疗研究，所采取的姑息治疗具体方法也未详细介绍，这使医生及相关人员产生较大困惑。我们应该加强对老年癌症患者的临床研究，加强对老年肿瘤综合评估的认识，掌握姑息治疗的评估方法和干预手段。使更多的老年肿瘤患者能从姑息治疗中获益。
